# The effect of fenugreek (*Trigonella foenum-graecum*) on stallion spermatozoa motility and vitality in vitro

**DOI:** 10.1007/s11259-026-11424-9

**Published:** 2026-07-24

**Authors:** Kateryna Vanivska, Ivana Kollárová, Ivana Mezeyová, Lucia Galovičová, Lucia Dianová, Marko Halo, Tomáš Slanina, Michal Lenický, Agnieszka Greń, Radovan Reisenauer, Peter Massányi

**Affiliations:** 1 Faculty of Biotechnology and Food Sciences, Institute of Applied Biology, Slovak University of Agriculture in Nitra, Trieda Andreja Hlinku 2, Nitra, 949 76 Slovak Republic; 2 Faculty of Horticulture and Landscape Engineering, Institute of Horticulture, Slovak University of Agriculture in Nitra, Trieda Andreja Hlinku 2, Nitra, 949 76 Slovak Republic; 3 Agrobiotech Research Centre, Slovak University of Agriculture in Nitra, Trieda Andreja Hlinku 2, Nitra, 949 76 Slovak Republic; 4 Faculty of Exact and Natural Sciences, Institute of Biology, University of the National Education Commission, Podchorążych 2, Krakow, 30-084 Poland

**Keywords:** *Trigonella foenum-graecum*, Spermatozoa, Motility, Viability, Antioxidants

## Abstract

**Abstract:**

This study evaluated the effects of *Trigonella foenum-graecum* L. (fenugreek) seed extract on stallion spermatozoa quality in vitro, focusing on motility, vitality, and functional competence. In vitro spermatozoa analyses showed that fenugreek seed extract did not demonstrate overt cytotoxicity under the tested conditions: S1 = 1562 µg/mL; S2 = 781 µg/mL; S3 = 390.5 µg/mL; S4 = 195.25 µg/mL; S5 = 97.62 µg/mL; S6 = 48.81 µg/mL; S7 = 24.40 µg/mL. Computer-assisted semen analysis (CASA) revealed a concentration- and time-dependent Treatment × Time interaction for several kinetic parameters. Total motility declined similarly across all groups over incubation time and was not significantly affected by extract concentration, whereas progressive motility, curvilinear velocity, beat-cross frequency, and amplitude of lateral head displacement were selectively increased at specific concentrations (mainly S4 and S7) at the last time point. Metabolic activity (MTT assay) increased significantly relative to the positive control across most concentrations, especially at early incubation time points, with fewer concentrations remaining elevated later. Sperm non-viability (eosin–nigrosin staining) and proAKAP4 levels were also significantly influenced by treatment in a non-monotonic manner: the highest concentration (S1) showed lower non-viability; similarly, the highest concentrations (S1–S3) showed reduced proAKAP4, whereas intermediate-to-lower concentrations showed higher non-viability (mainly S3, S4, S6, and S7) and increased proAKAP4 (mainly S4, S6, and S7), relative to the positive control. Physicochemical parameters of the extract (pH, osmolality) remained within physiological ranges, excluding osmotic or pH-related effects. Fenugreek seeds showed measurable antioxidant activity, with the ABTS assay showing the highest radical-scavenging capacity (10.67 ± 0.28 µmol Trolox equivalents/g dry weight). These findings indicate that fenugreek extract exerts selective, concentration- and time-dependent modulatory effects on stallion sperm function in vitro.

**Graphical abstract:**

**Figure:**
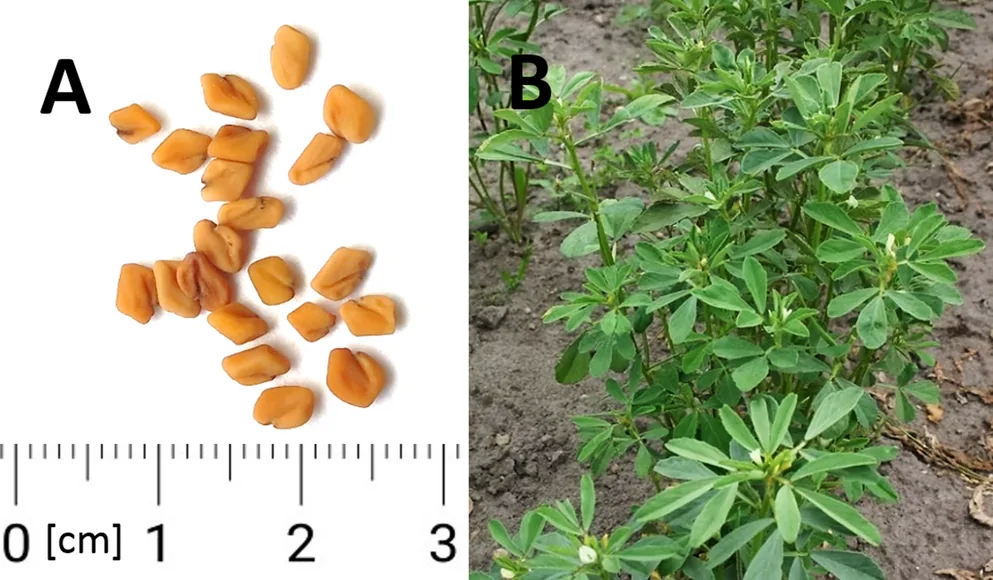

**Supplementary Information:**

The online version contains supplementary material available at https://doi.org/10.1007/s11259-026-11424-9.

## Introduction

*Trigonella foenum-graecum* L. (fenugreek) is an annual leguminous plant of the Fabaceae family. It is often used as a culinary ingredient and medicinal herb. Its biological activity is associated with a wide range of phytochemicals, including alkaloids, flavonoids, saponins, vitamins, and amino acids (Syed et al. 2020; Mohammadi et al. 2020).

Its bioactive constituents include compounds such as trigonelline, quercetin, kaempferol, and diosgenin. These compounds are known to have antioxidant, anti-inflammatory, and endocrine-related effects. They are involved in the regulation of several physiological processes, including reproductive function (Hilles and Mahmood 2021; Murria and Kaur 2018).

These compounds can also interact with reactive oxygen species (ROS). They help regulate oxidative processes and limit oxidative damage at the cellular level. Since oxidative stress plays an important role in sperm dysfunction, the antioxidant properties of fenugreek may be relevant for the protection of reproductive cells (Sasi et al. 2025; Wang et al. 2025).

Spermatozoa are highly sensitive to oxidative stress. This is primarily due to their limited endogenous antioxidant capacity (Wang et al. 2025). Another important factor is the high proportion of polyunsaturated fatty acids in their plasma membranes. This is especially evident in stallion spermatozoa, which are known to be sensitive to oxidative imbalance. Increased levels of ROS may lead to lipid peroxidation, reduced motility, mitochondrial dysfunction, and loss of membrane integrity. As a result, fertilizing capacity may be compromised (Peña 2020).

Previous studies have shown that natural antioxidants and plant extracts can influence sperm quality across different species. For example, polyphenol-rich extracts have been reported to improve antioxidant status and semen quality in rabbits (Vizzari et al. 2021). In addition, compounds such as caffeine and taurine have been shown to affect motility and preservation of stallion and avian spermatozoa under in vitro conditions (Slanina et al. 2018; Halo et al. 2023). These findings support the importance of oxidative balance in the regulation of sperm function (Walczak-Jedrzejowska et al. 2013).

Despite growing interest in fenugreek and its biological effects, its direct influence on sperm functional parameters remains insufficiently explored. This is particularly relevant in in vitro studies and in equine reproduction, where spermatozoa show high sensitivity to oxidative stress and require effective protective strategies (Sasi et al. 2025).

The aim of this study was to evaluate the in vitro effects of fenugreek (*Trigonella foenum-graecum*) seed extract on functional parameters of stallion spermatozoa, with a focus on motility, viability, metabolic activity, and sperm functional competence.

## Materials and methods

### Plant material

Aromatic and medicinal plant species *Trigonella foenum-graecum* L. (fenugreek) was used in this study. Seeds were purchased from the online store osiva-semena.sk (SemenaOnline, s.r.o.). The supplier confirmed that the seeds were suitable for outdoor cultivation as well as indoor cultivation as sprouts.

### Cultivation conditions

Fenugreek plants were cultivated at the Demonstration Garden of the Institute of Horticulture, Slovak University of Agriculture in Nitra, located in the Chrenová cadastral area at an elevation of 144 m a.s.l. (48°30′25″N, 18°10′08″E). The site lies within the warm Danubian Lowland region, characterized by loess hills with originally oak-cerro forests. The local climate is classified as mild and temperate, with annual average temperatures ranging between 8.28 °C and 10.05 °C, and precipitation between 529 mm and 895 mm (Hreško and Petluš 2015).

#### Cultivation and harvest

Fenugreek was sown directly into the soil on the field plot in rows spaced 0.4 × 0.1 m, with 5 rows sown in length of 4.5 m. No fertilizers, biostimulants or chemical plant protection were used during the plant cultivation. Drip irrigation Netafim Techline 1.6 LPH – litres per hour (Dripper Line) was applied as needed during the vegetation period to maintain optimal soil moisture monitored using the UNI-T UT333S device (Uni-Trend Technology (China) Co., Dongguan City, China). Weed control and soil aeration were carried out by hand hoeing. During the fenugreek harvest entire plants were manually harvested at the mature fruit stage, then dried on sieves at 25–30 °C in a well-ventilated room. Subsequently, seeds were manually extracted from the pods. The dry seeds of fenugreek were stored in plastic containers in darkness at room temperature, prepared for further processing and analysis.

### Extract preparation (methanol)

One gram of fenugreek seeds was homogenized (Heidolph Silent Crusher M, Heidolph Instruments, Schwabach, Germany) in 5 mL of 80% methanol (≥ 99.9%, suitable for HPLC, Sigma-Aldrich) in a test tube. This mixture was then placed on an orbital shaker (Biosan, PSU-10i) and extraction process started. Three extract replicates were prepared. After 48 h, the mixture was filtered, and the extract was stored at 4 °C for the analysis of antioxidant activity.

### Antioxidant activity assays

Antioxidant activity was evaluated in laboratories of AgroBioTech Research Centre in Nitra using DPPH (2,2-diphenyl-1-pikrylhydrazyl), ABTS (2,2′-azino-bis(3-ethylbenzothiazoline-6-sulfonic acid) and FRAP (Ferric Reducing Antioxidant Power) assays. Trolox (SigmaAldrich, Schnelldorf, Germany) served as the calibration standard for evaluating antioxidant activity which was expressed as µmol Trolox Equivalent per 1 g of dry weight (µmol TE/g DW).

### Extract preparation (ethanol)

One gram of fenugreek seeds was homogenized (Heidolph Silent Crusher M, Heidolph Instruments, Schwabach, Germany) in 5 mL of 80% ethanol (p.a. 96%, Centralchem) in a test tube. This mixture was then placed on an orbital shaker (Biosan, PSU-10i) to initiate the extraction process. After 48 h, the mixture was filtered, and the extract was stored at 4 °C for further analysis of its modulatory effects.

### Animals and semen collection

Ejaculate samples were obtained from three healthy adult breeding stallions of the Dutch Warmblood, Belgian Warmblood, and Belgian Sport Horse breeds (*n* = 3). The stallions were aged 6–12 years and maintained under standard conditions at the Institute of Animal Husbandry of the Faculty of Agrobiology and Food Resources, Slovak University of Agriculture in Nitra, Slovakia. All stallions were clinically healthy, with no prior reproductive interventions affecting the study.

Ejaculates were collected in the morning hours using an artificial vagina (Colorado model, Minitub, Tiefenbach, Germany). The stallions were stimulated by the presence of a mare in estrus (Halo et al. 2023). The artificial vagina was heated to 40–42 °C during collection. Semen collection was performed by mounting a phantom. Immediately after collection, the ejaculate was filtered through sterile gauze and subjected to laboratory examination (Tirpák et al. 2021).

All procedures were conducted in the laboratories of the Slovak University of Agriculture in Nitra, Faculty of Biotechnology and Food Sciences, Institute of Applied Biology.

### Experimental design

Each ejaculate was divided into treatment groups (S1–S7) and control groups (K+, K−) to evaluate the modulatory effect of fenugreek extract. The experimental unit was defined as a single ejaculate obtained from an individual stallion.

A total of 10 ejaculates were collected from three stallions (two stallions provided three ejaculates each, and one stallion provided four ejaculates). All ejaculates were processed under standardized experimental conditions and equally distributed across all experimental groups, resulting in 10 experimental units (*n* = 10) per group.

Sample size was determined based on semen availability and consistency with previous in vitro sperm studies. No specific inclusion or exclusion criteria were applied.

Physiological saline (0.9% NaCl) was used as a simplified incubation medium to minimize the influence of external biochemical components and to enable direct assessment of fenugreek extract effects on sperm function. The experimental design was intended for short-term in vitro exposure rather than long-term sperm preservation or fertilization procedures.

### Sample preparation and incubation

An 80% fenugreek (*Trigonella foenum-graecum*) extract was used as the stock solution for the preparation of all tested concentrations. To establish the working concentration range, a preliminary screening was performed based on computer-assisted semen analysis (CASA) of sperm motility during incubation (0–3 h). The concentration of 1562 µg/mL was identified as the highest level at which stallion spermatozoa maintained stable motility throughout the experimental period. At this level, the final ethanol concentration was 0.625% (v/v).

Lower concentrations were prepared by sequential 1:1 dilutions with physiological saline (0.9% NaCl), resulting in the following concentrations: S1 = 1562; S2 = 781; S3 = 390.5; S4 = 195.25; S5 = 97.62; S6 = 48.81; S7 = 24.40 µg/mL.

The positive control (K+) consisted of semen diluted with physiological saline (1:2, v/v).

The negative control (K−) was prepared by diluting ethanol in physiological saline to achieve the same final ethanol concentration (0.625%, v/v) as in the highest extract concentration, allowing separation between the effects of fenugreek extract and ethanol exposure on sperm motility.

Following dilution, samples were incubated at 37 °C under controlled laboratory conditions throughout the experiment. The pH and osmolality of the analyzed *Trigonella foenum-graecum* extract concentrations are provided in Supplementary Table S1.

### CASA analysis

Spermatozoa motility is a key indicator of ejaculate quality, assessed using Computer-Assisted Semen Analysis (CASA) (Choi et al. 2022). Spermatozoa analysis was performed at 0, 1, 2, and 3 h using a CASA system (SpermVision, Minitube, Germany) coupled with an Olympus BX51 microscope (Olympus, Japan). Diluted semen samples were placed into a preheated Makler counting chamber (10 μm depth) at 37 °C (Tirpák et al. 2022). Each sample was assessed once per concentration and time point.

Following the preparation of the experimental treatments and their mixing with the semen samples, the sperm concentration during the exposure phase varied between approximately 50 and 150 × 10⁶ cells/mL due to individual ejaculate differences. However, immediately prior to loading into the counting chamber for data recording, an aliquot of each sample was further diluted with physiological saline (0.9% NaCl) to a standardized working concentration approximately between 25 and 30 × 10⁶ cells/mL. This adjustment was applied uniformly across all experimental groups (K+, K−, S1–S7) and at all incubation time points. This step brought the final sperm density strictly within the optimal operational range recommended for the Makler counting chamber (Tirpák et al. 2022), which spans from 20 to 50 × 10⁶ cells/mL. This standardized approach prevented cell collisions, ensured high tracking accuracy by the SpermVision software, and eliminated sperm concentration as a confounding factor across groups.

The consistency of this working concentration was verified automatically by the CASA software at each measurement, confirming that the sperm density remained within the recommended limits for accurate motility assessment. For each sample, a minimum of 8 microscopic fields was recorded and analyzed, evaluating at least 200 spermatozoa in total to ensure a representative assessment. The CASA system settings, including the frame acquisition rate (FPS), were predefined by the SpermVision software according to the manufacturer’s recommendations for stallion semen analysis.

The evaluated parameters included total motility (MOT; %), progressive motility (PRO; %), curvilinear velocity (VCL; µm/s), amplitude of lateral head displacement (ALH; µm), and beat cross frequency (BCF; Hz) (Dianová et al. 2023).

### MTT assay

The MTT (methylthiazolyldiphenyl-tetrazolium bromide) assay is a colorimetric method based on the reduction of yellow MTT to purple formazan by mitochondrial dehydrogenases of metabolically active cells (Halo et al. 2021). The resulting formazan formation is proportional to cellular metabolic activity and viability. Absorbance is measured spectrophotometrically at 570 nm (Goodwin 2007).

Samples (100 µl) containing all tested concentrations were pipetted into a 96-well plate in triplicate. MTT solution (final concentration 5 mg/mL in physiological saline (0.9% NaCl; Braun, B. Braun Melsungen AG, Germany) was added at 20 µl per well. The plates were incubated at 37 °C for 1 h in the dark. After incubation, the reaction was terminated by adding 40 µl of isopropanol (Centralchem, Bratislava, Slovakia) to solubilize the formed formazan crystals. Absorbance was measured at 570 nm with a reference wavelength of 620 nm using an ELISA microplate reader (Multiscan FC, ThermoFisher Scientific, Finland). The results were expressed as relative absorbance, reflecting metabolic activity of the samples (Dianová et al. 2023).

### proAKAP4 analysis

ProAKAP4 concentration was determined using a commercially available ELISA kit (Horse 4MID^®^ BB Kit, SPQI S.A.S., Lille, France), following the manufacturer’s instructions. All samples, standards, and controls were analyzed in technical triplicate. The main steps of the assay are summarized below.

Fresh semen samples were processed under standard laboratory conditions and subjected to chemical lysis using R5 Horse Spermatozoa Lysis Buffer followed by dilution with R3 Dilution Buffer. The lysates were homogenized by vortexing to ensure complete cell disruption and were subsequently incubated at room temperature to stabilize the samples prior to analysis.

For the immunoassay, standards, controls, and prepared semen samples were loaded onto a 96-well microplate pre-coated with anti-proAKAP4 monoclonal antibodies. After incubation at room temperature under gentle agitation, the wells were washed with the provided washing buffer to remove unbound material. A specific detection antibody was then added, followed by a second incubation and washing step.

Color development was achieved using a chromogenic substrate solution, and the reaction was stopped after a defined incubation period using stop solution. Optical density was measured at 450 nm using a microplate reader (Multiscan FC, Thermo Fisher Scientific, Finland).

ProAKAP4 concentrations were calculated using a standard curve generated from known calibrators provided with the kit. Final values were normalized to sperm concentration and expressed as ng/mL.

### Eosin–nigrosin staining

Eosin–nigrosin staining is used to assess sperm viability by differentiating live and dead spermatozoa based on membrane integrity. Live spermatozoa remain unstained, whereas dead spermatozoa stain pink or red due to increased membrane permeability. Nigrosin provides background contrast for microscopic evaluation (Slanina et al. 2018; Kanna and Shetty 2025).

A 5 µl drop of stallion semen was mixed by vortexing with 4 µl of 2.5% eosin prepared in distilled water. After 60 s, 4 µl of 10% nigrosin (prepared in distilled water) was added, and after 3 s, the samples were smeared onto glass slides. After air-drying, 200 spermatozoa were evaluated using an Olympus BX 51 microscope (Olympus, Japan) at 60× magnification. Cells with permeable plasma membranes were stained pink, while those with intact membranes remained unstained. The percentage of eosin-positive (stained) spermatozoa, indicating cells with a damaged or permeable plasma membrane, was recorded as the outcome measure for each sample and time point (Slanina et al. 2018; Halo et al. 2021).

### Statistical analysis

All statistical analyses, including descriptive statistics (mean ± SD), two-way repeated-measures ANOVA, Dunnett’s post-hoc tests, partial eta-squared effect size calculations, and graphical outputs, were performed using GraphPad Prism 10.4.1 (GraphPad Software Inc., San Diego, CA, USA). For each outcome variable (MOT, PRO, VCL, BCF, ALH, MTT, proAKAP4, and eosin–nigrosin staining), differences among the nine experimental groups (K+, K−, S1–S7) across the four incubation time points (T0–T3) were evaluated using a two-way repeated-measures ANOVA (Treatment × Time). Each ejaculate was treated as a matched subject measured repeatedly over time within each treatment group (matching: stacked). To account for violations of the sphericity assumption, the Geisser–Greenhouse correction was applied, and the adjusted degrees of freedom were used for the main effect of Time. Where the Treatment × Time interaction or main effects were statistically significant, pairwise comparisons between each concentration (S1–S7) or the negative control (K−) and the positive control (K+) were performed using Dunnett’s multiple comparisons test with a family-wise significance threshold of alpha = 0.05. Partial eta-squared ($\:{{\upeta\:}}_{\mathrm{p}}^{2}$) values for the Treatment main effect were calculated using the Subject error term (i.e., $\:\frac{S{S}_{Treatment}}{\left[S{S}_{Treatment}+S{S}_{Subject}\right]}$), consistent with the error term used in the corresponding F-test for this effect, whereas $\:{{\upeta\:}}_{\mathrm{p}}^{2}$ values for the Time and Treatment × Time interaction effects were calculated using the Residual error term, consistent with their respective F-tests. The significance levels were defined as follows: **** = *P* < 0.0001; *** = *P* < 0.001; ** = *P* < 0.01; * = *P* < 0.05.

Prior to fitting the repeated-measures models, the normality of the raw data distribution within each of the nine treatment groups was assessed using the D’Agostino–Pearson omnibus, Anderson–Darling, Shapiro–Wilk, and Kolmogorov–Smirnov tests. Homogeneity of variance (homoscedasticity) and residual normality were assessed on the corresponding ordinary (non-repeated) two-way ANOVA model, using Spearman’s rank correlation between the absolute residuals and the predicted values of the model, and the same four normality tests applied to the residuals, respectively; these diagnostics are available only for the ordinary ANOVA model, whereas the repeated-measures model was used for all reported hypothesis tests.

The number of ejaculates (*n* = 10) was determined by semen availability from the three participating stallions, consistent with sample sizes in comparable in vitro sperm studies. A formal a priori or retrospective power calculation was not performed. However, the partial eta-squared values obtained from the ANOVA models, which were large ($\:{{\upeta\:}}_{\mathrm{p}}^{2}\:$≥ 0.26) for the parameters showing statistically significant Treatment effects (MTT, proAKAP4, sperm non-viability, and beat-cross frequency), suggest that the sample size (*n* = 10 ejaculates) was reasonably adequate to detect treatment effects of this magnitude at an alpha level of 0.05. Conversely, the medium effect sizes observed for total motility, progressive motility, curvilinear velocity, and amplitude of lateral head displacement ($\:{{\upeta\:}}_{\mathrm{p}}^{2}$ = 0.07–0.11) indicate that the study may have had limited power to detect smaller treatment effects for these kinetic parameters, and the corresponding non-significant Treatment main effects should be interpreted with this in mind.

To directly address the hierarchical structure of the data arising from the unequal distribution of ejaculates among the three donor stallions (see Animals and semen collection and Experimental design), a supplementary linear mixed-effects model (LMM) was fitted for each of the eight outcome variables using IBM SPSS Statistics for Windows, Version 32.0 (IBM Corp., Armonk, NY, USA), Linear Mixed Models procedure. Treatment (9 levels), Time (4 levels), and their interaction were specified as fixed effects. Stallion identity was specified as a random intercept to account for the between-animal component of variance. The repeated Time factor was modelled with a first-order autoregressive (AR1) covariance structure, with the repeated-measures subject defined as the unique combination of stallion, ejaculate, and Treatment, reflecting the fact that each ejaculate–treatment combination was measured repeatedly across the four incubation time points. Restricted maximum likelihood (REML) estimation was used throughout. This analysis was performed as a sensitivity check to determine whether accounting for the stallion-level random effect altered the fixed-effects conclusions obtained from the primary repeated-measures ANOVA reported above.

## Results

### Antioxidant activity of fenugreek seed extract

In this study, the antioxidant activity of fenugreek seeds was assessed using three complementary assays: DPPH, ABTS, and FRAP (see Supplementary Table S2). The ABTS assay showed the highest sensitivity among the applied methods. Antioxidant activity measured by the ABTS assay was 10.67 ± 0.28 µmol TE/g DW, while values obtained by the DPPH and FRAP assays were lower, 4.35 ± 0.19 and 2.67 ± 0.11 µmol TE/g DW, respectively.

### Total motility (MOT)

Two-way repeated-measures ANOVA revealed a statistically significant Treatment × Time interaction for total motility (F(24,243) = 2.671, *P* < 0.0001). The main effect of Time was highly significant (F(2.735, 221.6) = 471.8, *P* < 0.0001), whereas the main effect of Treatment was not significant (F(8,81) = 1.042, *P* = 0.412). Post-hoc comparisons (Dunnett’s test) did not identify statistically significant differences between K + and any tested concentration or the negative control at any incubation time point.

**Fig. 1:**
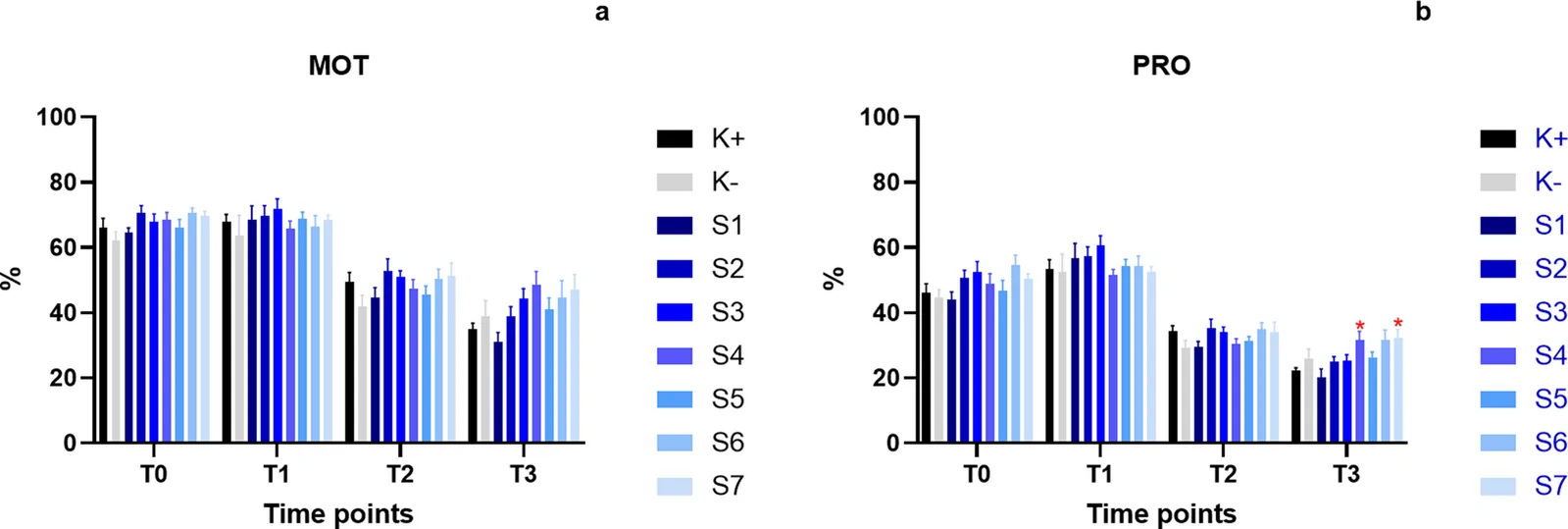
Total motility (MOT, %) (**a**) and progressive motility (PRO, %) (**b**) of stallion spermatozoa cultured with fenugreek extract at T0, T1, T2, and T3 of incubation. K + = positive control; K− = negative control; S1 = 1562 µg/mL; S2 = 781 µg/mL; S3 = 390.5 µg/mL; S4 = 195.25 µg/mL; S5 = 97.62 µg/mL; S6 = 48.81 µg/mL; S7 = 24.40 µg/mL. Statistical significance between tested concentrations and K + at each time point is indicated by * *P* < 0.05. Data were analyzed using two-way repeated-measures ANOVA (Treatment × Time) with Geisser–Greenhouse correction, followed by Dunnett’s multiple comparisons test; interaction P values are reported in the text

MOT results are presented in Fig. 1a.

### Progressive motility (PRO)

Two-way repeated-measures ANOVA revealed a statistically significant Treatment × Time interaction for progressive motility (F(24,243) = 2.042, *P* = 0.004). The main effect of Time was highly significant (F(2.633, 213.2) = 382.7, *P* < 0.0001), whereas the main effect of Treatment was not significant (F(8,81) = 1.282, *P* = 0.265). Post-hoc comparisons (Dunnett’s test) identified a statistically significant increase at T3 in S4 (mean difference = 9.267% points, adjusted *P* = 0.042) and S7 (mean difference = 9.896% points, adjusted *P* = 0.026) compared with K+.

PRO results are demonstrated in Fig. 1b.

Descriptive statistics (mean ± SD) of MOT and PRO at all incubation time points (T0–T3) are presented in Supplementary Tables 3 and 4, respectively.

### Curvilinear velocity (VCL)

Two-way repeated-measures ANOVA revealed a statistically significant Treatment × Time interaction for curvilinear velocity (F(24,243) = 4.237, *P* < 0.0001). The main effect of Time was highly significant (F(2.510, 203.3) = 279.0, *P* < 0.0001), whereas the main effect of Treatment was not significant (F(8,81) = 1.182, *P* = 0.320). Post-hoc comparisons using Dunnett’s test identified a statistically significant decrease at T2 for S1 (mean difference = 10.830 μm/s, adjusted *P* = 0.045) compared with K+. At T3, a statistically significant increase in VCL was observed compared with K + for S4 (mean difference = 16.020 μm/s, adjusted *P* = 0.006), S6 (mean difference = 15.860 μm/s, adjusted *P* = 0.046), and S7 (mean difference = 13.130 μm/s, adjusted *P* = 0.021).

**Fig. 2:**
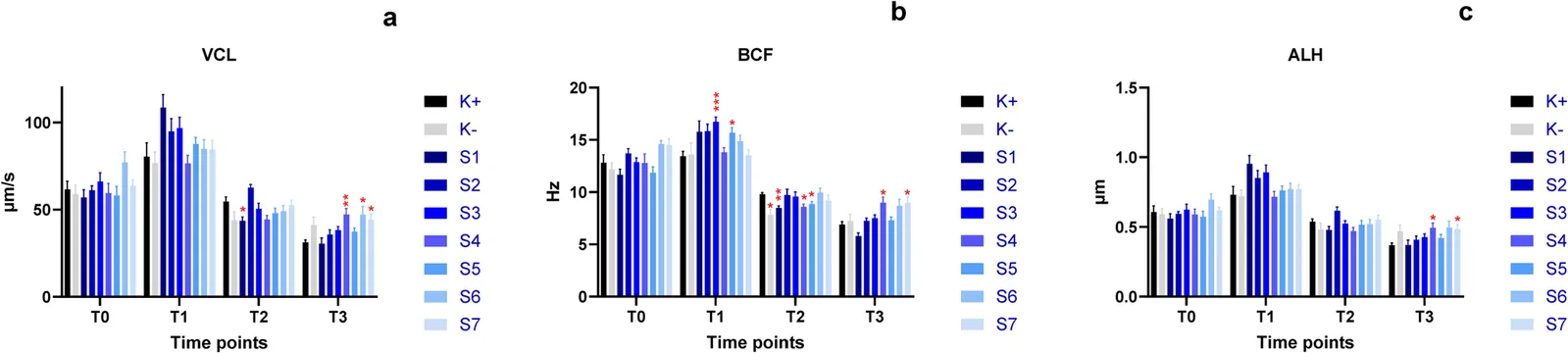
Curvilinear velocity (VCL, µm/s) (**a**), beat cross frequency (BCF, Hz) (**b**), and amplitude of lateral head displacement (ALH, µm) (**c**) of stallion spermatozoa cultured with fenugreek extract at T0, T1, T2, and T3 of incubation. K + = positive control; K− = negative control; S1 = 1562 µg/mL; S2 = 781 µg/mL; S3 = 390.5 µg/mL; S4 = 195.25 µg/mL; S5 = 97.62 µg/mL; S6 = 48.81 µg/mL; S7 = 24.40 µg/mL. Statistical significance between tested concentrations and K + at each time point is indicated by *** = *P* < 0.001; ** = *P* < 0.01; * = *P* < 0.05. Data were analyzed using two-way repeated-measures ANOVA (Treatment × Time) with Geisser–Greenhouse correction, followed by Dunnett’s multiple comparisons test; interaction P values are reported in the text

VCL results are reported in Fig. 2a.

### Beat cross frequency (BCF)

Two-way repeated-measures ANOVA revealed a statistically significant Treatment × Time interaction for beat-cross frequency (F(24,243) = 3.606, *P* < 0.0001). The main effect of Time was highly significant (F(2.618, 212.1) = 382.4, *P* < 0.0001), and the main effect of Treatment was also statistically significant (F(8,81) = 3.582, *P* = 0.001). Post-hoc comparisons (Dunnett’s test) identified a statistically significant decrease in K− compared to the K + at T2 (mean difference = 1.969 Hz, adjusted *P* = 0.028). At T1, among the tested concentrations, a statistically significant increase compared with K + was observed for S3 (mean difference = 3.303 Hz, adjusted *P* < 0.001) and S5 (mean difference = 2.262 Hz, adjusted *P* = 0.022). At T2, BCF showed a statistically significant decrease compared with K + for S1 (mean difference = 1.301 Hz, adjusted *P* = 0.001), S4 (mean difference = 1.199 Hz, adjusted *P* = 0.014), and S5 (mean difference = 0.929 Hz, adjusted *P* = 0.040). At T3, a statistically significant increase in BCF compared with K + was detected for S4 (mean difference = 2.108 Hz, adjusted *P* = 0.023) and S7 (mean difference = 2.090 Hz, adjusted *P* = 0.034).

BCF results are indicated in Fig. 2b.

### Amplitude of lateral head displacement (ALH)

Two-way repeated-measures ANOVA revealed a statistically significant Treatment × Time interaction for amplitude of lateral head displacement (F(24,243) = 4.058, *P* < 0.0001). The main effect of Time was highly significant (F(2.534, 205.3) = 245.3, *P* < 0.0001), whereas the main effect of Treatment was not significant (F(8,81) = 0.790, *P* = 0.613). Post-hoc comparisons (Dunnett’s test) identified a statistically significant increase at T3 in S4 (mean difference = 0.126 μm, adjusted *P* = 0.027) and S7 (mean difference = 0.116 μm, adjusted *P* = 0.047) compared with K+.

ALH results are shown in Fig. 2c.

Descriptive statistics (mean ± SD) of sperm kinematic parameters (VCL, BCF, ALH) at all incubation time points (T0–T3) are presented in Supplementary Tables 5, 6, and 7, respectively.

### Metabolic activity (MTT)

Two-way repeated-measures ANOVA revealed a statistically significant Treatment × Time interaction for metabolic activity (MTT assay) (F(24,243) = 7.036, *P* < 0.0001). Both main effects were statistically significant (Time: F(1.408, 114.0) = 136.5, *P* < 0.0001; Treatment: F(8,81) = 13.830, *P* < 0.0001). Post-hoc comparisons (Dunnett’s test) revealed that at T0, a significant increase compared with K + was observed for S1, S2, S3, S5, S6 and S7 (all *P* < 0.0001), as well as for S4 (*P* < 0.001). At T1, MTT remained significantly increased compared to K + for S1 (*P* < 0.001), S2 (*P* = 0.002), S3 (*P* = 0.006), S4 (*P* = 0.010), S5 (*P* = 0.043), and S6 (*P* = 0.004). This significant increase persisted at T2 for S1 (*P* = 0.005) and S6 (*P* = 0.034).

**Fig. 3:**
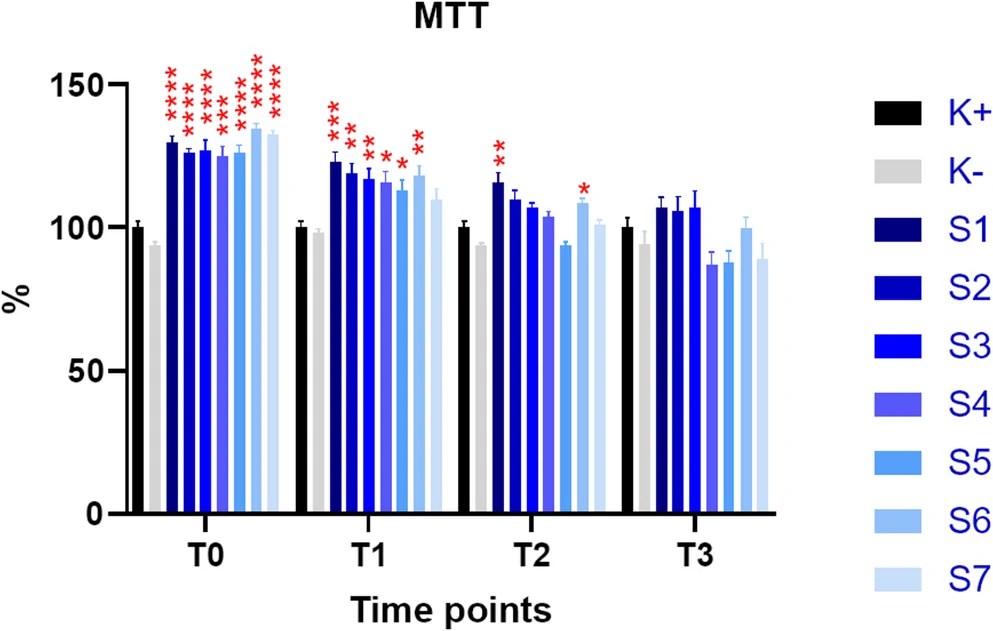
Metabolic activity (MTT, %) of stallion spermatozoa cultured with fenugreek extract at T0, T1, T2, and T3 of incubation. K + = positive control; K− = negative control; S1 = 1562 µg/mL; S2 = 781 µg/mL; S3 = 390.5 µg/mL; S4 = 195.25 µg/mL; S5 = 97.62 µg/mL; S6 = 48.81 µg/mL; S7 = 24.40 µg/mL. Statistical significance between tested concentrations and K + at each time point is indicated by **** = *P* < 0.0001; *** = *P* < 0.001; ** = *P* < 0.01; * = *P* < 0.05. Data were analyzed using two-way repeated-measures ANOVA (Treatment × Time) with Geisser–Greenhouse correction, followed by Dunnett’s multiple comparisons test; interaction P values are reported in the text

MTT results are recorded in Fig. 3.

Descriptive statistics (mean ± SD) of MTT at all incubation time points (T0–T3) are presented in Supplementary Table 8.

### Protein biomarker (proAKAP4)

Two-way repeated-measures ANOVA revealed a statistically significant Treatment × Time interaction for proAKAP4 concentration (F(24,243) = 13.180, *P* < 0.0001). Both main effects were statistically significant (Time: F(2.523, 204.3) = 248.5, *P* < 0.0001; Treatment: F(8,81) = 310.0, *P* < 0.0001). Post-hoc comparisons (Dunnett’s test) revealed a significant increase at T0 for S3 (*P* = 0.005), S4 (*P* < 0.001), and S7 (*P* < 0.001), as well as for S5 and S6 (both *P* < 0.0001). At T1, this increase remained significant for S4 (*P* = 0.032), S6 (*P* = 0.002), and S7 (*P* < 0.0001). By T2, while the significant increase persisted for S6 (*P* = 0.020) and S7 (*P* < 0.0001), a significant decrease had emerged for S1 (*P* < 0.001) and S2 (*P* = 0.003). T3 showed a significant decrease for S1, S2, and S3 (all *P* < 0.0001), and a significant increase for S4 (*P* = 0.002), S6 (*P* = 0.008), and S7 (*P* < 0.0001).

**Fig. 4:**
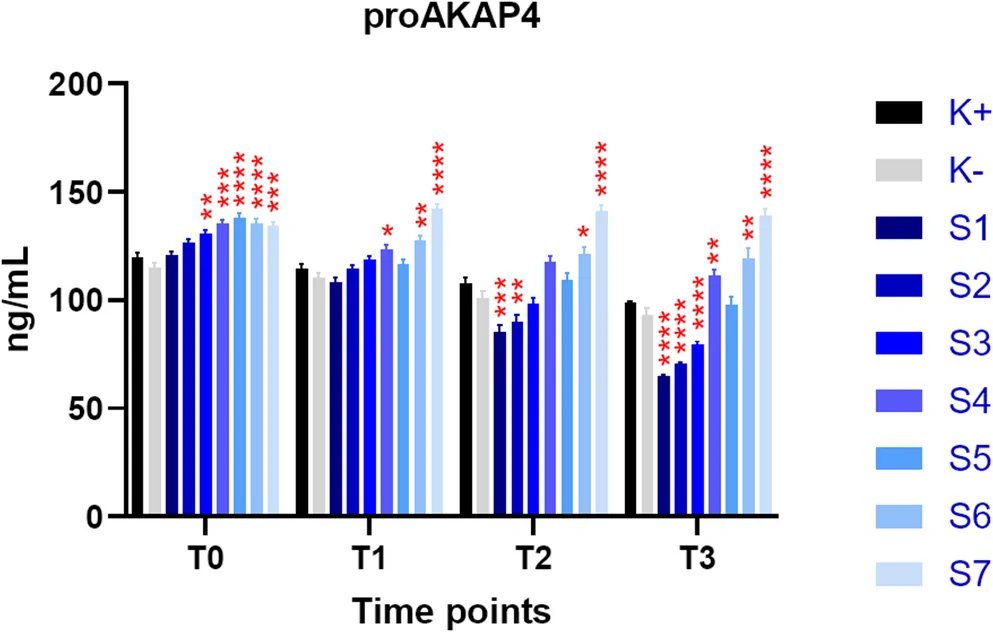
Protein biomarker (proAKAP4, ng/mL) of stallion spermatozoa cultured with fenugreek extract at T0, T1, T2, and T3 of incubation. K + = positive control; K− = negative control; S1 = 1562 µg/mL; S2 = 781 µg/mL; S3 = 390.5 µg/mL; S4 = 195.25 µg/mL; S5 = 97.62 µg/mL; S6 = 48.81 µg/mL; S7 = 24.40 µg/mL. Statistical significance between tested concentrations and K + at each time point is indicated by **** = *P* < 0.0001; *** = *P* < 0.001; ** = *P* < 0.01; * = *P* < 0.05. Data were analyzed using two-way repeated-measures ANOVA (Treatment × Time) with Geisser–Greenhouse correction, followed by Dunnett’s multiple comparisons test; interaction P values are reported in the text

ProAKAP4 results are shown in Fig. 4.

Descriptive statistics (mean ± SD) of proAKAP4 in stallion spermatozoa at all incubation time points (T0–T3) are presented in Supplementary Table 9.

### Sperm viability (eosin–nigrosin staining)

Two-way repeated-measures ANOVA revealed a statistically significant Treatment × Time interaction for sperm non-viability, expressed as the percentage of dead (eosin-positive) spermatozoa (F(24,243) = 2.465, *P* < 0.001). Both main effects were statistically significant (Time: F(1.485, 120.3) = 336.4, *P* < 0.0001; Treatment: F(8,81) = 277.2, *P* < 0.0001). Post-hoc comparisons (Dunnett’s test) revealed a significant decrease in the percentage of dead cells at T0 for K− (*P* < 0.0001) and S1 (*P* < 0.001), and a significant increase for S3, S4, and S7 (all *P* < 0.0001), as well as for S6 (*P* < 0.001). At T1, this pattern persisted, with a significant decrease for K− (*P* < 0.0001) and S1 (*P* < 0.001), and a significant increase for S3 and S4 (both *P* < 0.0001), S6 (*P* < 0.001), and S7 (*P* = 0.016). By T2, a significant decrease remained for K− (*P* < 0.0001), S1 (*P* < 0.001), and S5 (*P* = 0.006), while a significant increase persisted for S3, S4, and S7 (all *P* < 0.0001) and S6 (*P* = 0.002). At T3, the decrease remained significant for K− (*P* < 0.0001) and S1 (*P* = 0.006), while the increase remained significant for S4 and S7 (both *P* = 0.042) and S6 (*P* = 0.007).

**Fig. 5:**
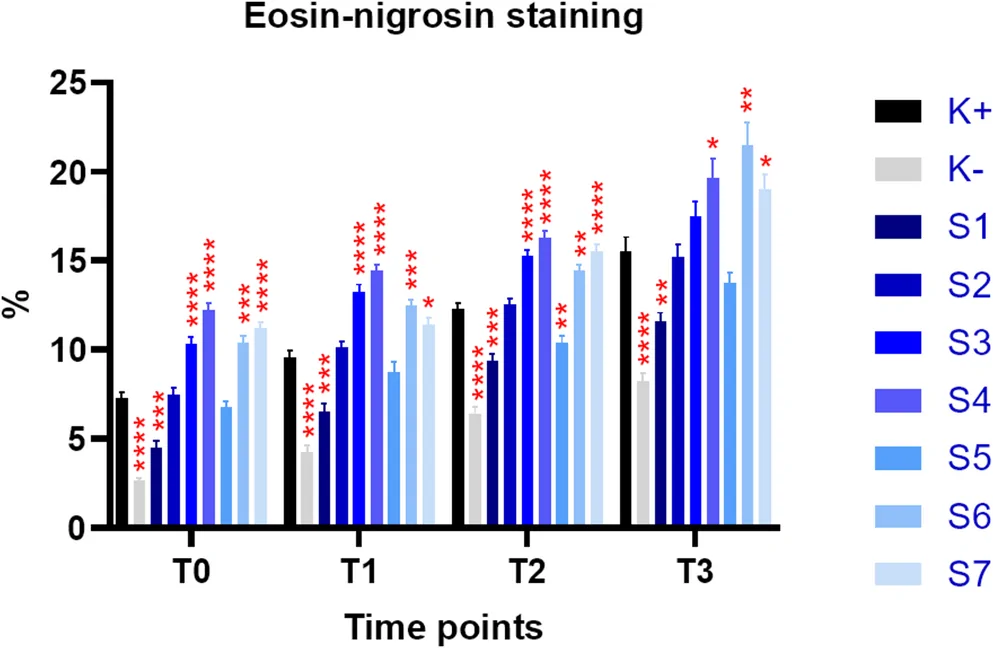
Sperm viability (eosin–nigrosin staining, %) of stallion spermatozoa cultured with fenugreek extract at T0, T1, T2, and T3 of incubation. The data represent the percentage of dead (eosin-positive) spermatozoa with a damaged plasma membrane (sperm non-viability). Data were analyzed using two-way repeated-measures ANOVA (Treatment × Time) with Geisser–Greenhouse correction, followed by Dunnett’s multiple comparisons test; the interaction P value is reported in the text. K + = positive control; K− = negative control; S1 = 1562 µg/mL; S2 = 781 µg/mL; S3 = 390.5 µg/mL; S4 = 195.25 µg/mL; S5 = 97.62 µg/mL; S6 = 48.81 µg/mL; S7 = 24.40 µg/mL. Statistical significance between tested concentrations and K + at each time point is indicated by **** = *P* < 0.0001; *** = *P* < 0.001; ** = *P* < 0.01; * = *P* < 0.05

Sperm viability results are presented in Fig. 5.

Descriptive statistics (mean ± SD) of sperm viability at all incubation time points (T0–T3) are presented in Supplementary Table 10.

Normality of the raw data and residuals, and homogeneity of variance (homoscedasticity), were assessed for all evaluated parameters; full diagnostic results are presented in Supplementary Table 11.

Results of the supplementary linear mixed-effects model, with stallion identity specified as a random intercept, are presented in Supplementary Table 12.

## Discussion

Several studies have investigated the effects of *Trigonella foenum-graecum* on reproductive parameters; however, most of them have focused on in vivo supplementation and systemic endocrine responses. In contrast, the present study examined the direct in vitro interaction between fenugreek seed extract and stallion spermatozoa, eliminating systemic influences and allowing assessment of sperm-specific cellular responses.

The antioxidant characterization of fenugreek seed extract confirmed measurable reducing capacity across DPPH, ABTS, and FRAP assays, with the highest activity observed in the ABTS assay (10.67 ± 0.28 µmol TE/g DW). This value is lower than those reported by Mekky et al. (2024), who observed TEAC levels of 35 ± 6 and 56.4 ± 0.7 µmol TE/g in different fenugreek cultivars, indicating considerable genotype-dependent variability in antioxidant potential.

Similarly, Lohvina et al. (2021) demonstrated that extraction conditions significantly influence antioxidant activity, with 70% and 96% ethanol extracts exhibiting stronger activity than lower ethanol concentrations (30% and 50%), based on the DPPH method. These findings are relevant to the present study given that ethanol was present in the experimental system at 0.625% (v/v), corresponding to the highest tested extract concentration. An ethanol-matched negative control was included to account for solvent-related effects. This control showed measurable effects on sperm parameters, suggesting that ethanol exposure may contribute to the observed responses.

Further literature supports the high variability of antioxidant capacity in fenugreek-derived materials. Bouhenni et al. (2021) reported an IC50 value of 343.75 µg/ml for fenugreek seed extract, while inhibition ranged from 35.84% to 82.57% depending on concentration. Çoban (2024) similarly demonstrated wide variation in antioxidant activity across 30 fenugreek genotypes, with DPPH inhibition ranging from 1.01% to 7.79% and ABTS values from 14.78% to 27.87%. Ghoora et al. (2020) reported TEAC values of 13.4 µmol TE/g (ABTS) and FRAP values of 10.0 µmol $\:F{e}^{2}+$/g in fenugreek microgreens, further highlighting the dependence of antioxidant expression on plant form (seeds versus sprouts), genotype, and extraction conditions.

Although fenugreek is widely recognized for its antioxidant potential, evidence regarding its direct effects on sperm function remains limited. Available studies indicate concentration-dependent responses in different species, where low concentrations may support sperm preservation, whereas higher concentrations can exert detrimental effects. In particular, İnanan et al. (2020) demonstrated that fenugreek seed extract at low concentrations (0.5%) preserved carp sperm motility by reducing oxidative stress during cold storage, whereas higher concentrations (2%) exerted detrimental effects. This dose-dependent response is consistent with the present findings, where stallion sperm functional parameters were modulated in a concentration- and time-dependent manner under in vitro incubation conditions.

At the cellular level, such effects may be associated with fenugreek-derived bioactive compounds, including flavonoids and saponins, which have been reported to exhibit antioxidant and membrane-modulating properties (Singh et al. 2022). Given the known sensitivity of spermatozoa to oxidative and membrane stress, the observed functional alterations are potentially associated with the antioxidant constituents and membrane-modulating properties of the extract, though the precise intracellular pathways remain to be verified.

Functionally, this interpretation is partly supported by the concentration-dependent changes observed in metabolic activity (MTT assay) and in sperm non-viability (eosin–nigrosin staining), both of which showed significant Treatment main effects in addition to the Treatment × Time interaction. Extract concentration significantly influenced proAKAP4 levels in a non-monotonic pattern. At later incubation stages (T2 and T3), the highest extract concentrations (S1–S3) were associated with a significant decrease in proAKAP4 concentration relative to the positive control, whereas lower concentrations (mainly S4, S6, and S7) were associated with a significant increase. This protein positively correlates with total and progressive motility in stallion spermatozoa (Dordas-Perpinyà et al. 2024). In our study, we observed a concurrent increase in proAKAP4 and selected kinetic traits (PRO, VCL, BCF, and ALH) at these same lower-to-intermediate concentrations (mainly S4 and S7) at the last time point. This trend is broadly consistent with the established correlation, although our in vitro design cannot prove a direct causal relationship between these endpoints. A comparable non-monotonic pattern was evident for sperm non-viability: the highest tested concentration (S1) was associated with a significant decrease in the proportion of non-viable spermatozoa relative to the positive control at every incubation time point, whereas intermediate-to-lower concentrations (mainly S3, S4, S6, and S7) were associated with a significant increase in this proportion. Overall, the evaluated parameters did not provide clear evidence of marked cytotoxic effects under the tested conditions, but rather a mixed, non-monotonic pattern of concentration-dependent changes that varied by parameter and time point.

The present data indicate a predominantly concordant, non-monotonic relationship between proAKAP4 concentration and sperm non-viability across treatment groups: both parameters were reduced at the highest extract concentrations and elevated at intermediate-to-lower concentrations, relative to the positive control. This pattern is inconsistent with the positive correlation typically reported between proAKAP4 concentration and sperm functional competence, since elevated proAKAP4 levels are generally associated with improved, rather than reduced, sperm quality. However, because we did not measure oxidative stress markers directly, this mechanistic interpretation remains speculative. This limitation is highly relevant given the distinct stallion sperm physiology, where energy for motility is strictly driven by mitochondrial oxidative phosphorylation rather than glycolysis (Gibb et al. 2014; Peña et al. 2019). Consequently, active mitochondrial metabolism inherently results in high physiological production of reactive oxygen species (ROS), which has been linked to normal sperm capacitation rather than functional impairment (Peña et al. 2019). Therefore, the relationship between oxidative stress and equine fertility is non-linear, as completely neutralizing ROS with antioxidants can inadvertently disrupt essential signaling pathways. This metabolic background is closely linked to proAKAP4—a flagellar structural protein and a key biomarker of equine fertility that positively correlates with total and progressive motility in stallion semen (Dordas-Perpinyà et al. 2024). Our study focused strictly on direct in vitro modulation, so these laboratory findings should be interpreted with caution. Since we did not evaluate actual fertility endpoints, we cannot directly link these isolated kinetic and molecular shifts to real equine reproductive outcomes. Further studies are needed to clarify their practical relevance.

To contextualize the practical relevance of these findings, partial eta-squared ($\:{{\upeta\:}}_{\mathrm{p}}^{2}$) effect sizes were calculated from the sums of squares of the repeated-measures ANOVA models, using the error term corresponding to the specific F-test for each effect (the Subject term for the Treatment main effect, and the Residual term for the Time and Treatment × Time effects), consistent with the actual denominator of the associated F-ratio. Following conventional benchmarks (small ≈ 0.01, medium ≈ 0.06, large ≥ 0.14; Cohen 1988), the Treatment main effect was medium for amplitude of lateral head displacement ($\:{{\upeta\:}}_{\mathrm{p}}^{2}\:$= 0.07), total motility ($\:{{\upeta\:}}_{\mathrm{p}}^{2}\:$= 0.09), curvilinear velocity ($\:{{\upeta\:}}_{\mathrm{p}}^{2}\:$= 0.10), and progressive motility ($\:{{\upeta\:}}_{\mathrm{p}}^{2}\:$= 0.11), consistent with the largely non-significant Treatment main effects observed for these kinetic traits (all *P* > 0.05) and the isolated nature of the corresponding post-hoc differences. In contrast, a large Treatment effect size was detected for beat-cross frequency ($\:{{\upeta\:}}_{\mathrm{p}}^{2}\:$= 0.26), metabolic activity ($\:{{\upeta\:}}_{\mathrm{p}}^{2}\:$= 0.58), proAKAP4 concentration ($\:{{\upeta\:}}_{\mathrm{p}}^{2}\:$= 0.97), and sperm non-viability ($\:{{\upeta\:}}_{\mathrm{p}}^{2}\:$= 0.96), which corresponded to statistically significant Treatment main effects for these parameters (all *P* ≤ 0.001). This pattern indicates that while the extract exerted a robust statistical influence on metabolic activity, sperm viability, proAKAP4 levels, and beat-cross frequency, the practical biological response with respect to the motility-related kinetic parameters was more modest and highly parameter-specific. Because total motility and progressive motility were not significantly improved by the Treatment main effect at any incubation time point, and the associated kinetic shifts identified by post-hoc comparisons were isolated to specific concentrations and time points, these findings do not support the direct application of fenugreek extract as a semen-preservation additive in equine reproduction at this stage. The lack of a consistent, linear dose–response pattern suggests that the practical biological relevance of these isolated kinetic changes remains limited under standard processing conditions, and further optimization is required before any extrapolation to applied semen-handling protocols such as cooled storage or extenders.

Taken together, fenugreek seed extract induced selective, concentration- and time-dependent modulation of sperm function, characterized by changes in selected kinetic parameters, metabolic activity, and sperm viability. However, as noted, many of these changes—particularly regarding kinetic parameters like PRO, VCL, BCF, and ALH—occurred as isolated effects at specific concentrations and time points rather than following a consistent, linear dose–response relationship. In addition, the experimental system included ethanol as a solvent component at the highest tested concentration, which showed measurable effects on sperm parameters. Therefore, the observed responses likely reflect a combined influence of fenugreek-derived compounds and solvent exposure, although their individual contributions cannot be fully separated under the present experimental design.

The present study has several methodological strengths, including a multi-parametric assessment of sperm function combining CASA, metabolic activity assays, sperm viability evaluation (expressed as the percentage of non-viable spermatozoa), and proAKAP4 quantification. This allowed assessment of sperm responses at multiple functional levels. However, several limitations should be considered when interpreting the results.

The number of donor stallions was limited to three, with repeated use of ejaculates across concentrations and incubation time points (see *Animals and semen collection*). This repeated-measures structure was explicitly accounted for in the statistical model, using a two-way repeated-measures ANOVA with Geisser–Greenhouse correction that treated each ejaculate as a matched subject; a retrospective effect-size-based assessment of the sample size is provided in the Statistical analysis section. Nonetheless, the limited number of individual stallions may still restrict the generalizability of the findings to the wider stallion population, and inter-individual biological variability between males could not be fully separated from treatment effects. To directly address the hierarchical (nested) structure of the data, in which ejaculates were unequally distributed among the three stallions, a supplementary linear mixed-effects model was fitted for each outcome variable (IBM SPSS Statistics, Linear Mixed Models procedure). Treatment, Time, and their interaction were specified as fixed effects, with a random intercept for individual stallion and a first-order autoregressive covariance structure for the repeated Time factor nested within ejaculate and Treatment (see Statistical analysis section for full model specification). Across all eight outcome variables, the Time effect and the Treatment × Time interaction remained statistically significant (*P* < 0.05) in the mixed model, matching the primary repeated-measures ANOVA. The Treatment main effect likewise remained consistent with the primary analysis for six of the eight parameters — statistically significant for BCF, sperm non-viability, proAKAP4, and MTT, and non-significant for VCL and ALH (full model statistics for all eight parameters are provided in Supplementary Table 12). This pattern did not hold for two kinetic parameters, however, for which the mixed model produced a different conclusion: the Treatment main effect, not significant in the primary ANOVA, reached statistical significance once the stallion-level random effect and the AR1 correlation structure were incorporated, for both total motility (MOT: *P* = 0.412 vs. *P* = 0.030) and progressive motility (PRO: *P* = 0.265 vs. *P* = 0.034). This shift likely reflects how the mixed model handles the data differently from the primary ANOVA. By isolating the variance associated with individual stallions and modelling the correlation between repeated measurements, the model provides a more precise estimate of the Treatment effect. Overall, therefore, the Time effect and the Treatment × Time interaction were fully consistent between the two models across all eight parameters, and the Treatment main effect was consistent for six of the eight, with a biologically plausible change in conclusion for MOT and PRO.

Thus, while the Time effect and the Treatment × Time interaction were unaffected by the choice of model, the Treatment main effect proved more sensitive to the modelling approach for two of the eight parameters than for the remaining six. However, the estimated variance component for the stallion-level random effect was small relative to its standard error for every parameter (e.g., total motility: variance = 45.8, SE = 47.0), indicating that the random-effect variance could not be estimated with reasonable precision given the small number of donor stallions (*n* = 3). This outcome is expected when so few clusters (stallions) are available for a random-effects model and should not be interpreted as evidence that inter-stallion variability is negligible; rather, it reflects a genuine statistical limitation of the current sample size at the stallion level, which could not be resolved through modelling alone (Harrison et al. 2018).

Formal assumption-checking (Supplementary Table 11) indicated that homoscedasticity was not met for most kinetic parameters — MOT, PRO, VCL, BCF, and ALH — nor for sperm non-viability, whereas it was confirmed for MTT and proAKAP4. Residual normality was confirmed only for PRO and ALH, whereas residuals of the remaining parameters (MTT, proAKAP4, sperm non-viability, MOT, VCL, and BCF) departed significantly from a Gaussian distribution. Given the balanced sample size (*n* = 10 per group across all treatment × time combinations), the potential impact of heteroscedasticity on Type I error control was likely reduced relative to an unbalanced design; however, equal group sizes do not fully eliminate this risk, as even balanced designs can show inflated Type I error rates under pronounced variance heterogeneity (Lix et al. 1996). Exact P values for the parameters showing significant heteroscedasticity (MOT, PRO, VCL, BCF, ALH, and sperm non-viability) should therefore be interpreted with appropriate caution. In contrast, residual non-normality is unlikely to have introduced substantial bias, given the well-documented robustness of ANOVA-based F-tests specifically to this violation (Schmider et al. 2010; Blanca et al. 2017).

Furthermore, the fenugreek extract was not chemically characterized in detail, and direct intracellular measurements of oxidative stress—such as reactive oxygen species (ROS) production, lipid peroxidation, malondialdehyde (MDA) levels, or antioxidant enzyme activities—were not performed in the treated spermatozoa. Consequently, while the extract demonstrated strong radical-scavenging capacity in cell-free assays (DPPH, ABTS, FRAP), any mechanistic interpretation regarding intracellular antioxidant pathways remains indirect and must be interpreted with caution. Finally, the use of a simplified saline-based incubation system enabled controlled evaluation of direct sperm–extract interactions but does not fully reproduce physiological conditions or standard semen extender environments, which may influence sperm responses in more complex systems.

Future studies should therefore include detailed phytochemical characterization of fenugreek extracts and direct assessment of oxidative stress pathways, including reactive oxygen species production, lipid peroxidation, and antioxidant enzyme activity. In addition, experiments using standard semen extenders or more physiologically relevant media would help to determine whether the observed effects persist under conditions closer to clinical or reproductive applications. A larger number of donor stallions is also recommended in future experimental designs: although a nested mixed-effects model incorporating individual stallion as a random effect was already applied in the present study, the small number of donor animals (*n* = 3) precluded a statistically reliable estimation of the stallion-level variance component. A larger cohort of stallions in future studies would allow this random effect to be estimated with adequate precision and would further help to partition donor-related variability from treatment effects.

In conclusion, *Trigonella foenum-graecum* seed extract exerts selective, concentration- and time-dependent modulatory effects on stallion spermatozoa under in vitro conditions. These effects are parameter-specific, frequently isolated, and do not follow a uniform or consistent dose-dependent pattern across all evaluated endpoints, which currently limits their direct practical application for semen preservation. While certain concentrations induced restricted shifts in specific kinetic and metabolic traits, the biological significance of these changes remains inconsistent. Therefore, under the present in vitro conditions, fenugreek extract should be regarded as a selective modulator of sperm function requiring deeper mechanistic validation rather than a uniformly beneficial agent.

## Supplementary information

Below is the link to the electronic supplementary material.

Supplementary Material 1 (EPS 56.5 KB)

Supplementary Material 2 (EPS 81.7 KB)

Supplementary Material 3 (EPS 21.1 KB)

Supplementary Material 4 (EPS 21.4 KB)

Supplementary Material 5 (EPS 21.7 KB)

Supplementary Material 6 (DOCX 14.9 KB)

Supplementary Material 7 (DOCX 14.1 KB)

Supplementary Material 8 (DOCX 15.1 KB)

Supplementary Material 9 (DOCX 79.0 KB)

Supplementary Material 10 (DOCX 15.5 KB)

Supplementary Material 11 (DOCX 15.7 KB)

Supplementary Material 12 (DOCX 15.4 KB)

Supplementary Material 13 (DOCX 15.5 KB)

Supplementary Material 14 (DOCX 15.6 KB)

Supplementary Material 15 (DOCX 15.7 KB)

Supplementary Material 16 (DOCX 15.3 KB)

Supplementary Material 17 (DOCX 15.8 KB)

## Data Availability

The datasets generated and/or analysed during the current study are available from the corresponding author on reasonable request.

## Declarations

### Research involving animals, their data or biological material

This study was conducted in accordance with national and institutional regulations governing the ethical use of animals in research. Ejaculated semen samples were obtained from clinically healthy breeding stallions during routine reproductive procedures, without any experimental manipulation or invasive intervention. Therefore, no ethical approval was required under the guidelines of the Slovak University of Agriculture in Nitra, Faculty of Biotechnology and Food Sciences, Institute of Applied Biology. All procedures complied with the principles outlined in the Basel Declaration and the International Association of Veterinary Editors’ Consensus Author Guidelines on Animal Ethics and Welfare.

## References

[CR1] Blanca MJ, Alarcón R, Arnau J et al (2017) Non-normal data: Is ANOVA still a valid option? Psicothema 4:552–557. 10.7334/psicothema2016.383 29048317

[CR2] Bouhenni H, Doukani K, Hanganu D et al (2021) Comparative analysis on bioactive compounds and antioxidant activity of Algerian fenugreek (*Trigonella foenum-graecum* L.) and Syrian cumin (Cuminum cyminum L.) seeds. Herba Pol 67:18–34. 10.2478/hepo-2021-0005

[CR3] Choi JW, Alkhoury L, Urbano LF et al (2022) An assessment tool for computer-assisted semen analysis (CASA) algorithms. Sci Rep 12:16830. 10.1038/s41598-022-20943-9 36207362 PMC9546881

[CR5] Çoban F (2024) Fenugreek Sprouts Around the World: Exploring Therapeutic and Nutritional Benefits. Food Sci Nutr 13:e4668. 10.1002/FSN3.4668 39803269 PMC11717055

[CR4] Cohen J (1988) Statistical Power Analysis for the Behavioral Sciences, 2nd edn. Lawrence Erlbaum Associates, Hillsdale, NJ

[CR6] Dianová L, Tirpák F, Halo M et al (2023) Effect of platinum nanoparticles on rabbit spermatozoa motility and viability. IJERR 32:270–277. 10.52756/IJERR.2023.V32.023

[CR7] Dordas-Perpinyà M, Yánez-Ortiz I, Sergeant N et al (2024) ProAKAP4 as Indicator of Long-Lasting Motility Marker in Post-Thaw Conditions in Stallions. Animals 14:1264. 10.3390/ani14091264 38731267 PMC11083937

[CR8] Ghoora MD, Haldipur AC, Srividya N (2020) Comparative evaluation of phytochemical content, antioxidant capacities and overall antioxidant potential of select culinary microgreens. J Agric Food Res 2:100046. 10.1016/J.JAFR.2020.100046

[CR9] Gibb Z, Lambourne SR, Aitken RJ (2014) The paradoxical relationship between stallion fertility and oxidative stress. Biol Reprod 91:77. 10.1095/biolreprod.114.118539 25078685

[CR10] Goodwin AM (2007) In vitro assays of angiogenesis for assessment of angiogenic and anti-angiogenic agents. Microvasc Res 74:172–183. 10.1016/J.MVR.2007.05.006 17631914 PMC2692317

[CR11] Halo M, Bułka K, Antos PA et al (2021) The effect of ZnO nanoparticles on rabbit spermatozoa motility and viability parameters in vitro. Saudi J Biol Sci 28:7450–7454. 10.1016/J.SJBS.2021.08.045 34867049 PMC8626300

[CR12] Halo M, Tirpák F, Slanina T et al (2023) A Combination of Taurine and Caffeine in Stallion Semen Extender Positively Affects the Spermatozoa Parameters. Cells 12:320. 10.3390/CELLS12020320 36672253 PMC9856288

[CR13] Harrison XA, Donaldson L, Correa-Cano ME et al (2018) A brief introduction to mixed effects modelling and multi-model inference in ecology. PeerJ 6:e4794. 10.7717/peerj.4794 29844961 PMC5970551

[CR14] Hilles AR, Mahmood S (eds) (2021) Fenugreek Biology and Applications. Springer Singapore

[CR15] Hreško J, Petluš P (eds) (2015) Atlas archetypov krajiny Slovenska. Univerzita Konštantína Filozofa v Nitre, Nitra

[CR16] İnanan BE, Kanyılmaz M, Makalesi A (2020) In Vitro Effects of Fenugreek, Sunflower, Green Cardamom and Seed Extracts on Motility Parameters and Oxidative Stress of Common Carp (Cyprinus carpio L.) spermatozoa. TURJAF 8:214–219. 10.24925/TURJAF.V8I1.214-219.2974

[CR17] Kanna S, Shetty A (2025) Eosin Nigrosin staining technique in assessment of sperm vitality in medical laboratories – A snippet from our experience on implementing the staining, interpretation and quality control procedures. IJOGR 10:227–229. 10.18231/J.IJOGR.2023.048

[CR18] Lix LM, Keselman JC, Keselman HJ (1996) Consequences of assumption violations revisited: A quantitative review of alternatives to the one-way analysis of variance F test. Rev Educ Res 4:579–619. 10.3102/00346543066004579

[CR19] Lohvina H, Sándor M, Wink M (2021) Effect of Ethanol Solvents on Total Phenolic Content and Antioxidant Properties of Seed Extracts of Fenugreek (*Trigonella foenum-graecum* L.) Varieties and Determination of Phenolic Composition by HPLC-ESI-MS. Diversity 14:7. 10.3390/D14010007

[CR20] Mekky RH, Abdel-Sattar E, Abdulla MH et al (2024) Metabolic profiling and antioxidant activity of fenugreek seeds cultivars ‘Giza 2’ and ‘Giza 30’ compared to other geographically-related seeds. Food Chem X 24:101819. 10.1016/J.FOCHX.2024.101819 39328377 PMC11426063

[CR21] Mohammadi M, Mashayekh T, Rashidi-Monfared S et al (2020) New insights into diosgenin biosynthesis pathway and its regulation in *Trigonella foenum-graecum* L. Phytochem Anal 31:229–241. 10.1002/PCA.2887 31469464

[CR22] Murria S, Kaur N (2018) Fenugreek alkaloids: A medicinal commodity. Asian J Soil Sci 13(2):13–14

[CR24] Peña FJ (2020) Molecular Biology of Spermatozoa. Int J Mol Sci 21:3060. 10.3390/IJMS21093060 32357538 PMC7246914

[CR23] Peña FJ, O’Flaherty C, Ortiz Rodríguez JM et al (2019) Redox Regulation and Oxidative Stress: The Particular Case of the Stallion Spermatozoa. Antioxidants 8:567. 10.3390/antiox8110567 31752408 PMC6912273

[CR25] Sasi SM, Alghoul NM, Prastiya RA, Salem K (2025) Effects of boiled fenugreek seed extract on testicular histology and reproductive parameters in adult male mice. Open Vet J 15:5041. 10.5455/OVJ.2025.V15.I10.22 41246404 PMC12613225

[CR26] Schmider E, Ziegler M, Danay E et al (2010) Is it really robust? Reinvestigating the robustness of ANOVA against violations of the normal distribution assumption. Methodology 4:147–151. 10.1027/1614-2241/a000016

[CR27] Singh N, Yadav SS, Kumar S, Narashiman B (2022) Ethnopharmacological, phytochemical and clinical studies on Fenugreek (*Trigonella foenum-graecum* L). Food Biosci 46:101546. 10.1016/J.FBIO.2022.101546

[CR28] Slanina T, Miškeje M, Tirpák F et al (2018) Caffeine strongly improves motility parameters of turkey spermatozoa with no effect on cell viability. Acta Vet Hung 66:137–150. 10.1556/004.2018.013 29580077

[CR29] Syed QA, Rashid Z, Ahmad MH et al (2020) Nutritional and therapeutic properties of fenugreek (*Trigonella foenum-graecum*): a review. Int J Food Prop 23:1777–1791. 10.1080/10942912.2020.1825482

[CR30] Tirpák F, Halo M, Tokárová K et al (2021) Composition of Stallion Seminal Plasma and Its Impact on Oxidative Stress Markers and Spermatozoa Quality. Life 11:1238. 10.3390/LIFE11111238 34833114 PMC8624310

[CR31] Tirpák F, Halo M, Tomka M et al (2022) Sperm Quality Affected by Naturally Occurring Chemical Elements in Bull Seminal Plasma. Antioxidants 11:1796. 10.3390/ANTIOX11091796/S1 36139870 PMC9495912

[CR32] Vizzari F, Massányi M, Knížatová N et al (2021) Effects of dietary plant polyphenols and seaweed extract mixture on male-rabbit semen: Quality traits and antioxidant markers. Saudi J Biol Sci 28:1017–1025. 10.1016/J.SJBS.2020.11.043 33424395 PMC7783798

[CR33] Walczak-Jedrzejowska R, Wolski JK, Slowikowska-Hilczer J (2013) The role of oxidative stress and antioxidants in male fertility. Cent Eur J Urol 66:60. 10.5173/CEJU.2013.01.ART19 PMC3921845 24578993

[CR34] Wang Y, Fu X, Li H (2025) Mechanisms of oxidative stress-induced sperm dysfunction. Front Endocrinol (Lausanne) 16:1520835. 10.3389/FENDO.2025.1520835/FULL 39974821 PMC11835670

