# Supplementary material for: The effect of fenugreek (Trigonella foenum-graecum) on stallion spermatozoa motility and vitality in vitro
Source: Vet Res Commun. 2026 Jul 24;50(5):482. doi: 10.1007/s11259-026-11424-9 (PMC13400685; doi:10.1007/s11259-026-11424-9)
Supplement: Supplementary file 6 — Supplementary Material 6 (DOCX 14.9 KB) [file 11259_2026_11424_MOESM6_ESM.docx]

**Supplementary Table 1.** pH values and osmolality of fenugreek extract concentrations

| **Sample label** | **Concentration of Fenugreek extract** (µg/mL) | **pH** | **Osmolality** **[mOsmol.kg^-1^]** |
| --- | --- | --- | --- |
| **K+** | semen + 0.9% NaCl, 1:2 v/v | 6.00 | 289 |
| **K-** | semen + 0.625% v/v ethanol | 6.06 | 396 |
| **S1** | 1562 | 5.75 | 338 |
| **S2** | 781 | 5.65 | 312 |
| **S3** | 390.5 | 5.54 | 299 |
| **S4** | 195.25 | 5,50 | 292 |
| **S5** | 97.62 | 5,48 | 289 |
| **S6** | 48.81 | 5,51 | 287 |
| **S7** | 24.40 | 5.55 | 286 |
