# Supplementary material for: The effect of fenugreek (Trigonella foenum-graecum) on stallion spermatozoa motility and vitality in vitro
Source: Vet Res Commun. 2026 Jul 24;50(5):482. doi: 10.1007/s11259-026-11424-9 (PMC13400685; doi:10.1007/s11259-026-11424-9)
Supplement: Supplementary file 7 — Supplementary Material 7 (DOCX 14.1 KB) [file 11259_2026_11424_MOESM7_ESM.docx]

**Supplementary Table 2.** Evaluation of fenugreek seed antioxidant activity

| **Plant material** | **Antioxidant activity (µmol TE/g DW)** | | |
| --- | --- | --- | --- |
|  | **DPPH** | **ABTS** | **FRAP** |
| **Fenugreek seeds** | 4.35 ± 0.19 | 10.67 ± 0.28 | 2.67 ± 0.11 |

DW - dry weight, TE - Trolox equivalent, values are means ± standard deviation
