# Supplementary material for: The effect of fenugreek (Trigonella foenum-graecum) on stallion spermatozoa motility and vitality in vitro
Source: Vet Res Commun. 2026 Jul 24;50(5):482. doi: 10.1007/s11259-026-11424-9 (PMC13400685; doi:10.1007/s11259-026-11424-9)
Supplement: Supplementary file 8 — Supplementary Material 8 (DOCX 15.1 KB) [file 11259_2026_11424_MOESM8_ESM.docx]

**Supplementary Table 3.** Descriptive statistics (mean ± SD) of stallion sperm motility (MOT) at all incubation time points (T0–T3)

| **Concentration** | **MOT** | | | |
| --- | --- | --- | --- | --- |
|  | **T0** | **T1** | **T2** | **T3** |
| **K+** | 66.23 ± 8.62 | 68.05 ± 6.93 | 49.43 ± 9.34 | 35.00 ± 5.63 |
| **K−** | 62.29 ± 8.20 | 63.73 ± 19.30 | 41.86 ± 11.13 | 39.00 ± 14.85 |
| **S1** | 64.53 ± 4.72 | 68.44 ± 13.83 | 44.72 ± 9.39 | 30.97 ± 9.39 |
| **S2** | 70.51 ± 7.38 | 69.85 ± 9.47 | 52.83 ± 11.83 | 38.81 ± 9.64 |
| **S3** | 67.78 ± 7.85 | 71.74 ± 10.24 | 51.13 ± 5.58 | 44.23 ± 9.94 |
| **S4** | 68.55 ± 7.05 | 65.85 ± 7.21 | 47.31 ± 9.17 | 48.49 ± 13.32 |
| **S5** | 66.21 ± 7.52 | 68.88 ± 6.43 | 45.75 ± 7.85 | 41.14 ± 10.73 |
| **S6** | 70.72 ± 4.46 | 66.32 ± 11.03 | 50.35 ± 9.49 | 44.53 ± 16.98 |
| **S7** | 69.59 ± 4.78 | 68.53 ± 4.18 | 51.35 ± 12.56 | 47.19 ± 14.49 |
