# Supplementary material for: The effect of fenugreek (Trigonella foenum-graecum) on stallion spermatozoa motility and vitality in vitro
Source: Vet Res Commun. 2026 Jul 24;50(5):482. doi: 10.1007/s11259-026-11424-9 (PMC13400685; doi:10.1007/s11259-026-11424-9)
Supplement: Supplementary file 9 — Supplementary Material 9 (DOCX 79.0 KB) [file 11259_2026_11424_MOESM9_ESM.docx]

**Supplementary Table 4.** Descriptive statistics (mean ± SD) of stallion sperm progressive motility (PRO) at all incubation time points (T0–T3)

| **Concentration** | **PRO** | | | |
| --- | --- | --- | --- | --- |
|  | **T0** | **T1** | **T2** | **T3** |
| **K+** | 46.30 ± 8.12 | 53.44 ± 8.96 | 34.36 ± 5.32 | 22.31 ± 2.57 |
| **K−** | 44.65 ± 7.48 | 52.34 ± 17.79 | 29.13 ± 7.63 | 25.81 ± 9.78 |
| **S1** | 44.02 ± 7.31 | 56.64 ± 14.70 | 29.55 ± 5.32 | 20.21 ± 8.26 |
| **S2** | 50.70 ± 7.37 | 57.20 ± 9.60 | 35.41 ± 8.31 | 25.10 ± 4.62 |
| **S3** | 52.37 ± 10.50 | 60.66 ± 9.45 | 34.11 ± 4.87 | 25.41 ± 5.51 |
| **S4** | 48.78 ± 10.09 | 51.50 ± 5.76 | 30.36 ± 5.28 | 31.58 ± 8.60* |
| **S5** | 46.67 ± 10.39 | 54.41 ± 6.27 | 31.35 ± 4.40 | 26.22 ± 5.55 |
| **S6** | 54.57 ± 9.65 | 54.28 ± 10.04 | 35.09 ± 5.98 | 31.61 ± 9.93 |
| **S7** | 50.34 ± 5.05 | 52.42 ± 5.63 | 34.06 ± 9.76 | 32.20 ± 8.41* |

Statistical significance is indicated as follows: * = p < 0.05
