# Supplementary material for: The effect of fenugreek (Trigonella foenum-graecum) on stallion spermatozoa motility and vitality in vitro
Source: Vet Res Commun. 2026 Jul 24;50(5):482. doi: 10.1007/s11259-026-11424-9 (PMC13400685; doi:10.1007/s11259-026-11424-9)
Supplement: Supplementary file 10 — Supplementary Material 10 (DOCX 15.5 KB) [file 11259_2026_11424_MOESM10_ESM.docx]

**Supplementary Table 5.** Descriptive statistics (mean ± SD) stallion sperm kinematic parameter (VCL) at all incubation time points (T0–T3)

| **Concentration** | **VCL** | | | |
| --- | --- | --- | --- | --- |
|  | **T0** | **T1** | **T2** | **T3** |
| **K+** | 61,65 ± 14,96 | 80,36 ± 25,20 | 54,50 ± 9,02 | 31,20 ± 4,64 |
| **K−** | 58,95 ± 16,50 | 76,66 ± 20,17 | 43,82 ± 15,77 | 41,10 ± 14,64 |
| **S1** | 56,92 ± 14,41 | 108,60 ± 23,51 | 43,68 ± 6,95* | 30,48 ± 10,43 |
| **S2** | 61,13 ± 8,13 | 94,98 ± 23,04 | 62,55 ± 6,08 | 35,76 ± 8,58 |
| **S3** | 66,13 ± 15,87 | 96,78 ± 19,64 | 50,48 ± 10,06 | 38,29 ± 6,64 |
| **S4** | 59,47 ± 17,75 | 76,42 ± 14,98 | 44,35 ± 7,21 | 47,22 ± 10,80** |
| **S5** | 58,17 ± 16,46 | 87,61 ± 12,23 | 47,91 ± 9,33 | 37,40 ± 6,86 |
| **S6** | 77,16 ± 19,29 | 84,87 ± 16,25 | 49,17 ± 9,94 | 47,05 ± 14,93* |
| **S7** | 63,64 ± 10,97 | 84,45 ± 16,42 | 52,61 ± 8,92 | 44,33 ± 10,65* |

Statistical significance is indicated as follows: ** = p < 0.01; * = p < 0.05
