# Supplementary material for: The effect of fenugreek (Trigonella foenum-graecum) on stallion spermatozoa motility and vitality in vitro
Source: Vet Res Commun. 2026 Jul 24;50(5):482. doi: 10.1007/s11259-026-11424-9 (PMC13400685; doi:10.1007/s11259-026-11424-9)
Supplement: Supplementary file 11 — Supplementary Material 11 (DOCX 15.7 KB) [file 11259_2026_11424_MOESM11_ESM.docx]

**Supplementary Table 6.** Descriptive statistics (mean ± SD) stallion sperm kinematic parameter (BCF) at all incubation time points (T0–T3)

| **Concentration** | **BCF** | | | |
| --- | --- | --- | --- | --- |
|  | **T0** | **T1** | **T2** | **T3** |
| **K+** | 12,80 ± 2,45 | 13,42 ± 1,48 | 9,79 ± 0,57 | 6,88 ± 0,91 |
| **K−** | 12,18 ± 2,03 | 13,59 ± 3,51 | 7,82 ± 1,69* | 7,23 ± 2,03 |
| **S1** | 11,66 ± 1,70 | 15,77 ± 3,27 | 8,49 ± 0,65** | 5,79 ± 0,93 |
| **S2** | 13,69 ± 1,51 | 15,83 ± 2,14 | 9,72 ± 1,79 | 7,24 ± 0,86 |
| **S3** | 12,88 ± 1,27 | 16,72 ± 1,45*** | 9,57 ± 1,39 | 7,50 ± 1,05 |
| **S4** | 12,77 ± 2,79 | 13,82 ± 1,40 | 8,59 ± 0,88* | 8,99 ± 1,71* |
| **S5** | 11,85 ± 1,76 | 15,68 ± 1,55* | 8,86 ± 0,77* | 7,29 ± 0,98 |
| **S6** | 14,60 ± 1,05 | 14,86 ± 1,83 | 9,94 ± 1,36 | 8,69 ± 1,97 |
| **S7** | 14,48 ± 1,98 | 13,51 ± 1,81 | 9,18 ± 1,69 | 8,97 ± 1,82* |

Statistical significance is indicated as follows: *** = P < 0.001; ** = p < 0.01; * = p < 0.05
