# Supplementary material for: The effect of fenugreek (Trigonella foenum-graecum) on stallion spermatozoa motility and vitality in vitro
Source: Vet Res Commun. 2026 Jul 24;50(5):482. doi: 10.1007/s11259-026-11424-9 (PMC13400685; doi:10.1007/s11259-026-11424-9)
Supplement: Supplementary file 12 — Supplementary Material 12 (DOCX 15.4 KB) [file 11259_2026_11424_MOESM12_ESM.docx]

**Supplementary Table 7.** Descriptive statistics (mean ± SD) of stallion sperm kinematic parameter (ALH) at all incubation time points (T0–T3)

| **Concentration** | **ALH** | | | |
| --- | --- | --- | --- | --- |
|  | **T0** | **T1** | **T2** | **T3** |
| **K+** | 0,61 ± 0,14 | 0,73 ± 0,19 | 0,54 ± 0,06 | 0,37 ± 0,05 |
| **K−** | 0,59 ± 0,13 | 0,72 ± 0,15 | 0,48 ± 0,15 | 0,47 ± 0,15 |
| **S1** | 0,56 ± 0,11 | 0,95 ± 0,19 | 0,48 ± 0,08 | 0,37 ± 0,12 |
| **S2** | 0,59 ± 0,06 | 0,85 ± 0,17 | 0,62 ± 0,09 | 0,41 ± 0,09 |
| **S3** | 0,62 ± 0,13 | 0,89 ± 0,17 | 0,52 ± 0,07 | 0,43 ± 0,08 |
| **S4** | 0,59 ± 0,13 | 0,72 ± 0,12 | 0,47 ± 0,08 | 0,49 ± 0,11* |
| **S5** | 0,57 ± 0,12 | 0,76 ± 0,10 | 0,52 ± 0,10 | 0,42 ± 0,08 |
| **S6** | 0,70 ± 0,13 | 0,77 ± 0,14 | 0,52 ± 0,11 | 0,50 ± 0,15 |
| **S7** | 0,62 ± 0,08 | 0,77 ± 0,11 | 0,55 ± 0,10 | 0,48 ± 0,11* |

Statistical significance is indicated as follows: * = p < 0.05
