# Supplementary material for: The effect of fenugreek (Trigonella foenum-graecum) on stallion spermatozoa motility and vitality in vitro
Source: Vet Res Commun. 2026 Jul 24;50(5):482. doi: 10.1007/s11259-026-11424-9 (PMC13400685; doi:10.1007/s11259-026-11424-9)
Supplement: Supplementary file 13 — Supplementary Material 13 (DOCX 15.5 KB) [file 11259_2026_11424_MOESM13_ESM.docx]

**Supplementary Table 8.** Descriptive statistics (mean ± SD) of metabolic activity (MTT) of stallion spermatozoa at all incubation time points (T0–T3)

| **Concentration** | **MTT** | | | |
| --- | --- | --- | --- | --- |
|  | **T0** | **T1** | **T2** | **T3** |
| **K+** | 100,00 ± 6,56 | 100,00 ± 6,80 | 100,00 ± 6,80 | 100,00 ± 10,90 |
| **K-** | 94,00 ± 3,39 | 98,00 ± 5,00 | 94,00 ± 2,03 | 94,07 ± 14,67 |
| **S1** | 130,00 ± 6,50**** | 122,90 ± 11,11*** | 116,00 ± 10,01** | 107,10 ± 11,11 |
| **S2** | 126,30 ± 3,89**** | 118,90 ± 11,14** | 110,00 ± 9,82 | 105,70 ± 15,82 |
| **S3** | 127,00 ± 11,13**** | 117,00 ± 11,30** | 107,00 ± 4,96 | 107,00 ± 18,38 |
| **S4** | 125,00 ± 10,73*** | 116,00 ± 11,34* | 104,00 ± 4,96 | 87,15 ± 13,62 |
| **S5** | 126,30 ± 7,48**** | 113,00 ± 11,44* | 93,90 ± 3,76 | 87,78 ± 13,21 |
| **S6** | 134,70 ± 5,13**** | 118,00 ± 11,42** | 108,50 ± 5,11* | 99,89 ± 11,82 |
| **S7** | 132,70 ± 3,82**** | 110,00 ± 11,42 | 101,00 ± 4,97 | 88,99 ± 17,23 |

Statistical significance is indicated as follows: **** = P < 0.0001; *** = P < 0.001; ** = P < 0.01; * = P < 0.05
