# Supplementary material for: The effect of fenugreek (Trigonella foenum-graecum) on stallion spermatozoa motility and vitality in vitro
Source: Vet Res Commun. 2026 Jul 24;50(5):482. doi: 10.1007/s11259-026-11424-9 (PMC13400685; doi:10.1007/s11259-026-11424-9)
Supplement: Supplementary file 14 — Supplementary Material 14 (DOCX 15.6 KB) [file 11259_2026_11424_MOESM14_ESM.docx]

**Supplementary Table 9.** Descriptive statistics (mean ± SD) of sperm protein biomarker (proAKAP4) in stallion spermatozoa at all incubation time points (T0–T3)

| **Concentration** | **proAKAP4** | | | |
| --- | --- | --- | --- | --- |
|  | **T0** | **T1** | **T2** | **T3** |
| **K+** | 120,11 ± 5,78 | 114,70 ± 6,38 | 107,60 ± 9,08 | 98,94 ± 1,59 |
| **K-** | 115,30 ± 5,85 | 110,30 ± 6,49 | 101,30 ± 9,24 | 93,18 ± 9,47 |
| **S1** | 120,70 ± 5,97 | 108,30 ± 6,48 | 85,54 ± 9,43*** | 65,20 ± 1,58**** |
| **S2** | 126,50 ± 5,99 | 114,40 ± 6,47 | 90,22 ± 8,82** | 70,89 ± 1,82**** |
| **S3** | 130,60 ± 5,84** | 118,50 ± 6,45 | 98,30 ± 9,11 | 79,78 ± 3,43**** |
| **S4** | 135,20 ± 5,79*** | 123,50 ± 6,12* | 117,70 ± 9,01 | 111,70 ± 7,39** |
| **S5** | 138,30 ± 5,95**** | 116,60 ± 6,31 | 109,60 ± 9,43 | 97,85 ± 11,05 |
| **S6** | 135,80 ± 5,64**** | 127,40 ± 6,53** | 121,60 ± 9,43* | 119,40 ± 14,45** |
| **S7** | 134,20 ± 5,72*** | 142,40 ± 6,62**** | 141,00 ± 9,04**** | 139,30 ± 9,57**** |

Statistical significance is indicated as follows: **** = P < 0.0001; *** = P < 0.001; ** = P < 0.01; * = P < 0.05
