# Supplementary material for: The effect of fenugreek (Trigonella foenum-graecum) on stallion spermatozoa motility and vitality in vitro
Source: Vet Res Commun. 2026 Jul 24;50(5):482. doi: 10.1007/s11259-026-11424-9 (PMC13400685; doi:10.1007/s11259-026-11424-9)
Supplement: Supplementary file 15 — Supplementary Material 15 (DOCX 15.7 KB) [file 11259_2026_11424_MOESM15_ESM.docx]

**Supplementary Table 10.** Descriptive statistics (mean ± SD) of sperm non-viability (percentage of dead spermatozoa evaluated by eosin–nigrosin staining) at all incubation time points (T0–T3)

| **Concentration** | **Eosin-nigrosin staining** | | | |
| --- | --- | --- | --- | --- |
|  | **T0** | **T1** | **T2** | **T3** |
| **K+** | 7,27 ± 1,06 | 9,55 ± 1,29 | 12,30 ± 1,02 | 15,50 ± 2,61 |
| **K-** | 2,67 ± 0,40**** | 4,27 ± 1,12**** | 6,39 ± 1,19**** | 8,25 ± 1,31**** |
| **S1** | 4,52 ± 1,10*** | 6,52 ± 1,39*** | 9,40 ± 1,19*** | 11,63 ± 1,40** |
| **S2** | 7,50 ± 1,16 | 10,13 ± 1,08 | 12,52 ± 1,19 | 15,25 ± 2,06 |
| **S3** | 10,33 ± 1,13**** | 13,27 ± 1,25**** | 15,27 ± 1,03**** | 17,50 ± 2,48 |
| **S4** | 12,25 ± 1,10**** | 14,45 ± 1,13**** | 16,30 ± 1,08**** | 19,65 ± 3,43* |
| **S5** | 6,75 ± 1,19 | 8,74 ± 1,82 | 10,42 ± 1,12** | 13,75 ± 1,80 |
| **S6** | 10,39 ± 1,29*** | 12,49 ± 1,03*** | 14,44 ± 1,06** | 21,50 ± 3,94** |
| **S7** | 11,22 ± 1,06**** | 11,43 ± 1,10* | 15,50 ± 1,24**** | 19,00 ± 2,57* |

Statistical significance is indicated as follows: **** = P < 0.0001; *** = P < 0.001; ** = P < 0.01; * = P < 0.05
