# Supplementary material for: The effect of fenugreek (Trigonella foenum-graecum) on stallion spermatozoa motility and vitality in vitro
Source: Vet Res Commun. 2026 Jul 24;50(5):482. doi: 10.1007/s11259-026-11424-9 (PMC13400685; doi:10.1007/s11259-026-11424-9)
Supplement: Supplementary file 16 — Supplementary Material 16 (DOCX 15.3 KB) [file 11259_2026_11424_MOESM16_ESM.docx]

**Supplementary Table 11.** Normality and homoscedasticity diagnostics (D'Agostino–Pearson, Anderson–Darling, Shapiro–Wilk, Kolmogorov–Smirnov, and Spearman's rank correlation tests) for raw data and model residuals across all evaluated sperm parameters (MOT, PRO, VCL, BCF, ALH, MTT, proAKAP4, and eosin–nigrosin staining), based on the ordinary two-way ANOVA model.

| **Parameter** | **Spearman rs** | **Spearman P** | **Homoscedasticity** | **Residual normality** |
| --- | --- | --- | --- | --- |
| **MOT (total motility)** | -0.212 | <0.0001 | Failed | Failed (all 4) |
| **PRO (progressive motility)** | 0.257 | <0.0001 | Failed | Passed (all 4) |
| **VCL (curvilinear velocity)** | 0.299 | <0.0001 | Failed | Failed (all 4) |
| **BCF (beat-cross frequency)** | 0.188 | 0.0002 | Failed | Failed (all 4) |
| **ALH (lateral head displacement)** | 0.155 | 0.0016 | Failed | Passed (all 4) |
| **MTT (viability)** | -0.055 | 0.150 | Passed | Failed (all 4) |
| **proAKAP4** | 0.069 | 0.095 | Passed | Failed (all 4) |
| **Eosin–Nigrosin staining (viability)** | 0.344 | <0.0001 | Failed | Failed (all 4) |
