# Supplementary material for: The effect of fenugreek (Trigonella foenum-graecum) on stallion spermatozoa motility and vitality in vitro
Source: Vet Res Commun. 2026 Jul 24;50(5):482. doi: 10.1007/s11259-026-11424-9 (PMC13400685; doi:10.1007/s11259-026-11424-9)
Supplement: Supplementary file 17 — Supplementary Material 17 (DOCX 15.8 KB) [file 11259_2026_11424_MOESM17_ESM.docx]

**Supplementary Table 12**. Fixed-effects results (Type III tests) and estimated stallion-level random-effect variance from the supplementary linear mixed-effects model (Treatment × Time, random intercept for stallion, first-order autoregressive [AR1] covariance structure for repeated Time within ejaculate and Treatment), fitted separately for each outcome variable using IBM SPSS Statistics (Linear Mixed Models procedure; restricted maximum likelihood estimation).

| **Parameter** | **Treatment F (df), P** | **Time F (df), P** | **Treatment × Time F (df), P** | **Var (Stallion), SE** |
| --- | --- | --- | --- | --- |
| MOT | 2.256 (8, 90.1), 0.030 | 271.631 (3, 227.3), <0.001 | 2.370 (24, 227.3), <0.001 | 45.80, SE 46.95 |
| PRO | 2.199 (8, 99.6), 0.034 | 277.408 (3, 226.4), <0.001 | 1.848 (24, 226.4), 0.012 | 17.20, SE 18.02 |
| VCL | 1.546 (8, 106.9), 0.150 (ns) | 243.875 (3, 232.0), <0.001 | 4.268 (24, 232.0), <0.001 | 17.84, SE 20.54 |
| BCF | 4.151 (8, 114.6), <0.001 | 327.414 (3, 228.8), <0.001 | 3.556 (24, 228.8), <0.001 | 0.078, SE 0.112 |
| ALH | 1.096 (8, 99.3), 0.373 (ns) | 220.614 (3, 230.9), <0.001 | 4.354 (24, 230.9), <0.001 | 0.002, SE 0.002 |
| Non-viability | 194.905 (8, 105.8), <0.001 | 392.110 (3, 206.1), <0.001 | 2.785 (24, 206.1), <0.001 | 0.077, SE 0.097 |
| proAKAP4 | 196.870 (8, 164.9), <0.001 | 355.490 (3, 239.0), <0.001 | 18.565 (24, 239.0), <0.001 | 1.591, SE 1.928 |
| MTT | 12.714 (8, 81.9), <0.001 | 96.295 (3, 214.1), <0.001 | 5.564 (24, 214.1), <0.001 | 0.395, SE 1.893 |

*ns = not significant (P > 0.05). Var (Stallion) = estimated variance of the random intercept for stallion identity; SE = standard error of that variance estimate. MOT = total motility; PRO = progressive motility; VCL = curvilinear velocity; BCF = beat-cross frequency; ALH = amplitude of lateral head displacement; Non-viability = percentage of eosin-positive (dead) spermatozoa (eosin–nigrosin staining); proAKAP4 = protein biomarker concentration; MTT = metabolic activity.*
